# RHOA in Gastric Cancer: Functional Roles and Therapeutic Potential

**DOI:** 10.3389/fgene.2019.00438

**Published:** 2019-05-15

**Authors:** Seungyoon Nam, Jung Ho Kim, Dae Ho Lee

**Affiliations:** ^1^Department of Genome Medicine and Science, College of Medicine, Gachon University, Incheon, South Korea; ^2^Gachon Institute of Genome Medicine and Science, Gachon University Gil Medical Center, Incheon, South Korea; ^3^Gachon Advanced Institute of Health Sciences and Technology, Gachon University, Incheon, South Korea; ^4^Department of Life Sciences, Gachon University, Seongnam, South Korea; ^5^Division of Gastroenterology, Department of Internal Medicine, Gachon University Gil Medical Center, School of Medicine, Gachon University, Incheon, South Korea; ^6^Gachon Medical Research Institute, Gachon University Gil Medical Center, Incheon, South Korea; ^7^Department of Internal Medicine, Gachon University Gil Medical Center, Incheon, South Korea; ^8^Department of Internal Medicine, Gachon University College of Medicine, Incheon, South Korea

**Keywords:** RHOA, gastric cancer, stomach cancer, therapeutics, functions

## Abstract

The well-known signal mediator and small GTPase family member, RHOA, has now been associated with the progression of specific malignancies. In this review, we appraise the biomedical literature regarding the role of this enzyme in gastric cancer (GC) signaling, suggesting potential clinical significance. To that end, we examined RHOA activity, with regard to second-generation hallmarks of cancer, finding particular association with the hallmark “activation of invasion and metastasis.” Moreover, an abundance of studies show RHOA association with Lauren classification diffuse subtype, in addition to poorly differentiated GC. With regard to therapeutic value, we found RHOA signaling to influence the activity of specific widely used chemotherapeutics, and its possible antagonism by various dietary constituents. We also review currently available targeted therapies for GC. The latter, however, showed a paucity of such agents, underscoring the urgent need for further investigation into treatments for this highly lethal malignancy.

## Introduction

The RHO GTPase enzyme family, including RHOA, Rac, and cdc42, is essential for diverse biological processes, including cell morphology phenotypes, cell polarity, and cell migration, in diverse cancer types (Etienne-Manneville and Hall, 2002; Hanna and El-Sibai, 2013; Lin and Zheng, 2015; Haga and Ridley, 2016; Woldu et al., 2018). Recently, it was shown that RHOA can be targeted by small molecule inhibitors, in cancer, implicating it as a potential druggable target (Shang et al., 2012; Chang et al., 2016a). Due to a scarcity of RHOA reviews in the field of gastric cancer (GC), our focus herein is limited to RHOA, and its related GTPase family members (Etienne-Manneville and Hall, 2002; Heasman and Ridley, 2008; Hanna and El-Sibai, 2013; Haga and Ridley, 2016; Woldu et al., 2018) in GC.

**Figure 1:**
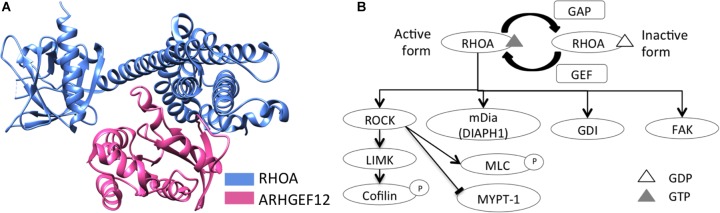
RHOA schematic structure and its downstream signaling. **(A)** Schematic structure of RHOA and a GEF protein, LARG (ARHGEF12), were represented (RCSB PDB ID: 1X86). **(B)** RHOA downstream signaling was represented, regarding actin reorganization, cell motility, and cell migration.

Structural domains of RHO GTPase family members include a downstream effector protein-binding, and a GTP-/GDP-binding, domain (Hanna and El-Sibai, 2013). RHOA, like other RHO GTPases, is regulated by guanine nucleotide-exchange factors (GEFs), GTPase-activating proteins (GAPs), and guanine nucleotide-dissociation inhibitors (GDIs) (Hanna and El-Sibai, 2013). RHOA activation, by GEFs, facilitates its binding to GTP, as well as its release of GDP (Heasman and Ridley, 2008). Activated RHOA then recruits downstream effector proteins, including ROCK, LIMK, MLC, cofilin, PKN1, MYPT-1, and mammalian homolog of diaphanous (mDia) (Schwartz, 2004; Hanna and El-Sibai, 2013; Prudnikova et al., 2015), and it is involved in actin reorganization, cell motility, and cell migration (Hanna and El-Sibai, 2013; Prudnikova et al., 2015) (Figure 1). As opposed to GEFs, GAPs inactivate various RHO-GTPase forms (e.g., RHOA-GDP) (Hanna and El-Sibai, 2013). Finally, GDIs interact with RHOA-GDP complexes to sequester RHOA from membranes, thus suppressing their activation (Dovas and Couchman, 2005; Knezevic et al., 2007).

Recently, roles for RHOA in cell motility have been identified, in many diverse types of cancer (Prudnikova et al., 2015), but RHOA’s clinical significance (including therapeutic feasibility), to GC, one of the most lethal cancers in East Asia (Jemal et al., 2011), remains little known. Herein, the functional roles of RHOA, and its clinical relevance to GC, are systematically reviewed throughout the literature. Specifically, we find that RHOA is a strong potential druggable target, as well as a biomarker candidate, for GC, which currently lacks effective targeted therapies.

## RHOA Functions and Its Biological Behavior in GC

### RHOA Functions in Second-Generation Cancer Hallmarks

First, we explored literature regarding RHOA, in gastric cancer, in PubMed, using the search terms, “RHOA,” “expression,” and “cancer,” as of March 6, 2018, resulting in 1,536 articles. Subsequently, we manually inspected the presence of term “gastric” in the article titles to secure RHOA’s relevance to GC, resulting in 63 publications. Then, we co-searched the term “RHOA” with the terms in the ten 2nd-generation hallmark phrases (Hanahan and Weinberg, 2011) in abstracts and main texts of the 63 publications. We extracted 47 most relevant articles, regarding hallmarks of the cancer. These 47 were the basis for this section (Supplementary Figure S1). Along with the 47, manually selected publications were also reviewed in this section.

Recently, a second generation of cancer hallmarks was proposed (Hanahan and Weinberg, 2011), including: (1) activation of invasion and metastasis; (2) resistance to cell death; (3) sustainment of proliferative signaling; (4) evasion of growth suppressors; (5) dysregulation of cellular energetics; (6) replicative immortality; (7) angiogenesis; (8) genome instability and mutation; (9) tumor-promoting inflammation; and (10) avoidance of immune destruction. Using these, we assigned RHOA functions, from the 47 identified publications, according to relevance to the 10 cancer hallmarks. These 47 were then aligned to the three hallmark terms: activation of invasion and metastasis (term #1, above); resistance to cell death (term #2); and sustainment of proliferative signaling (term #3) (Supplementary Figure S2). The other cancer hallmark terms were not revealed in these 47 publications, but we strongly believe that RHOA signaling associates with all 10.

As shown in Supplementary Figure S2, most of the publications were assigned to term #1, “activation of invasion and metastasis.” In GC, this hallmark term associates with RHOA, in signal pathways (Chiou et al., 2001; Murray et al., 2008; Bennett et al., 2011; Zhou et al., 2011; Gomes et al., 2013). During GC cancer cell migration, a sialylated glycan antigen, Sialyl-Lewis X (SLe^x^, synthesized by ST3GAL4), is often expressed on cell surfaces (Gomes et al., 2013). Through ST3GAL4 expression, leading to SLe^x^ biosynthesis, activation of RHOA, as well as c-Met, associates with SLe^x^-induced GC invasion phenotypes (Gomes et al., 2013). In recent studies (Murray et al., 2008; Bennett et al., 2011), lysophosphatidic acid (LPA), through Neuroepithelial Cell Transforming 1 (NET1, a protein upregulated in GC tissues) (Leyden et al., 2006), activates RHOA, leading to cell invasion and migration. We especially noted that *NET1* mRNA expression was upregulated in gastric epithelia infected by *Helicobacter pylori* (*H. pylori*), the primary risk factor for gastric carcinogenesis (Chiou et al., 2001).

One proposed mechanism of tumorigenesis, via *H. pylori* infection of gastric epithelial cells, with regard to cell migration, is that CagA (cytotoxin-associated gene A product), secreted during *H. pylori* activation of RHOA, through SHP-2 (encoded by *PTPN11*), actuates a Raf/Mek/ERK signaling cascade (Hagymasi and Tulassay, 2014). Moreover, we found RHOA to be involved in LPA-induced transcription of the metastasis-associated urokinase-type plasminogen activator receptor (uPAR, encoded by *PLAUR*), resulting in GC cell invasion, through an unknown mechanism (Kim et al., 2008). In another study, LPA-induced RHOA activity was suppressed by cross-linked hyaluronic acid gel, in a GC cell line (AGS), thereby inhibiting GC cell migration (Lan et al., 2016). While we could find no literature regarding germline *RHOA* mutations, a somatic mutation, RHOA-G17V, has been reported to positively associate with peripheral T-cell lymphoma chemoresponse (Manso et al., 2014).

Activating invasion by RHOA in GC is also mediated by CXCL12, a ligand for CXCR4, leading to activation of RHOA, Rac, and Cdc42 through mTOR signaling (Chen et al., 2012). In fact, rapamycin, an inhibitor of mTOR signaling, suppressed GC cell migration induced by CXCL12, indicating mTOR signaling as a possible therapeutic target in GC (Chen et al., 2012). Moreover, GC cell motility was induced by the C5a receptor (CD88), in association with activated RHOA (Kaida et al., 2016), while more recently, RHOA’s role, in activating invasion, was revealed to be epigenetically regulated by the non-coding RNA, miR-31, potentially targeting *RHOA*, in MKN-45 GC cells (Korourian et al., 2017b).

Growth factors can also induce GC cell invasion and migration. For example, TGFβ1 signaling activated RHOA, leading to scirrhous GC cell migration, via the epithelial-to-mesenchymal transition (EMT) (Shinto et al., 2010). More specifically, in scirrhous GC cell lines, RHOA activity was successfully repressed by an antagonist of the TGFβ receptor type I (encoded by *TGFBR1*), a mediator of TGFβ1 signaling (Shinto et al., 2010). Additionally, RHOA activity was reported as promoted by the maternal embryonic leucine zipper kinase (gene symbol, *MELK*), in GC cell migration (Du et al., 2014).

Dietary constituents can also regulate GC cell migration and invasion, through RHOA suppression (Ho et al., 2011). For example, benzyl isothiocyanate (BITC), an isothiocyanate found in mustards, repressed both *RHOA* and *FAK* mRNAs, inhibiting migration of AGS GC cells (Ho et al., 2011). RHOA also closely aligned with ROCK, which regulated invasion of OCUM-2MD3, a scirrhous GC cell line (Matsuoka et al., 2011).

Another dietary constituent, of watercress, phenethyl isothiocyanate (PEITC), downregulated AGS GC cell migration, through RHOA activity inhibition, leading to suppression of the metastasis-promoting urokinase-type plasminogen activator (UPA), cyclooxygenase-2 (COX-2), inducible nitric oxide synthase (iNOS), and NF-κB (Yang et al., 2010). A constituent of numerous plants, gallic acid, also suppressed RHOA activity, and that of the GTPases Cdc42, and Rac1, leading to inhibition of AGS GC cell migration (Ho et al., 2010). The flavonoid nobiletin, isolated from citrus fruit peels, was similarly reported to inhibit FAK/Ras enzymatic activity, downregulating RHOA/Cdc42/Rac1 protein expression, to subsequently inhibit AGS GC cell migration (Lee et al., 2011). We note that the study of dietary agents associated with reduced cancer risk, by identifying their potentially antineoplastic constituents in treatment of cultured cancer cells, is an essential first preclinical step (Yang et al., 2016). However, this must be then translated to animals, disease models, etc., prior to any remote possibility of use in humans (Cherng et al., 2007).

Epigenetically, GC cell invasion was suppressed by the non-coding RNA, miR-647, through a RHOA-mediated SRF/MYH9 axis (Ye et al., 2017), while miR-29, in association with chemotherapy, inhibited GC cell invasion and migration, *in vitro* and *in vivo* (Wang et al., 2015).

The cancer hallmark term, “resistance to cell death” (#2 above), also highly associated with RHOA. While a role for RHOA in apoptosis remains unresolved in GC (Cai et al., 2008), evidence does exist for apoptotic effects of RHOA/Rock signal pathway inhibition, in GC (Cai et al., 2008; Xu et al., 2012). One recent report showed that RHOA activation, in association with cell detachment-induced apoptosis (i.e., anoikis, cell death due to loss of cell-extracellular matrix contacts), resulted in enhanced assembly of actin filaments and focal adhesions (Cai et al., 2008). Also, resistance to chemotherapy-induced apoptosis (Kaufmann and Earnshaw, 2000), in GC cells, was reported to be mediated by RHOA activation (Kang et al., 2005). Activation of RHOA and NF-κB, by *H. pylori* infection, induced plasminogen activator inhibitor-2 (PAI-2; SERPINB2), leading to inhibition of apoptosis in gastric epithelial cells (Varro et al., 2004).

The cancer hallmark term, “sustainment of proliferative signaling” (hallmark #3 above), has yet to be clearly linked to GC, with specific regard to RHOA (Ghosh et al., 1999). However, a few studies have implicated RHOA as playing roles in GC cell proliferation. For example, one study showed that RHOA inhibition suppressed GC cell growth, albeit with lack of a proposed molecular mechanism (Liu et al., 2004). Also, when RHOA was inhibited in the GC cells, via siRNA, G1/S progression was slowed, through upregulation of the INK4 family cell cycle inhibitors, p15^INK4b^ (*CDKN2B*), p16^INK4a^ (*CDKN2A*), p18^INK4c^ (*CDKN2C*), and p19^INK4d^ (*CDKN2B*). These events were postulated as mediated by RHOA/Rock pathway inhibition (Zhang et al., 2009), and resulted in inhibition of CDK4 and CDK6 activity. Also, p21^CIP1^ (*CDKN1A*) and p27^KIP1^ (*CDKN1B*), cell cycle inhibitors of CDK2, were upregulated through a RHOA/mDia pathway, during RHOA suppression (Zhang et al., 2009). However, the detailed mechanisms of this phenomenon remain unknown.

The tumor microenvironment (e.g., stromal cells, cancer-associated fibroblasts, etc.) also plays an important role in multiple cancer hallmarks (Hanahan and Weinberg, 2011). In tumor microenvironment, tumor stroma interacting with cancer cells support tumor growth and progression, and include heterogeneous cell types (fibroblasts, myofibroblasts, endothelial cells, macrophages, diverse immune cells, and extracellular matrix (ECM)) (Tevis et al., 2017). Spheroids can mimic these multicellular nature and ECM, while monolayer system is too simplified to represent the interaction of a growing tumor and stroma (Tevis et al., 2017). Moreover, it is believed that distinct regions of the microenvironment comprise a cancer stem cell (CSC) “niche” (Plaks et al., 2015). Also, tumor-derived spheroids are used to purpose for the enrichment of CSCs or stem-like cells (Ishiguro et al., 2017). In one CSC assay, spheroid formation (Zhao et al., 2015), RHOA was hyperactivated in spheroid GC cells, compared to monolayer GC cell colonies of diffuse type GC cells (Yoon et al., 2016). Another stemness phenotype, drug expulsion by the membrane transporter P-glycoprotein, was also found to be attenuated by RHOA pharmacological inhibition (Pinzon-Daza et al., 2014). These findings may implicate RHOA signaling in the promotion of CSC phenotypes (Yoon et al., 2016).

Aberrant post-transcriptional events may also contribute to regulation of RHOA signaling. For example, recently, a contradictory role for RHOA, in two Lauren diffuse type GC cell lines (HSC-59, GSU) of 17 GC cell lines (12 for diffuse type and five for intestinal type), was suggested, in that low RHOA protein expression, due to aberrant alternative splicing of *RHOA* transcripts, was found in the two GC cell lines (HSC-59, GSU) (Miyamoto et al., 2018).

GC cell lines ranges in diverse histology, Lauren classification, *RHOA* mutation statuses, and RHOA protein expression (Supplementary Table S1). Thus, different GC cell line characteristics may impact on RHOA function, and GC cell line studies above need to be carefully interpreted.

**Figure 2:**
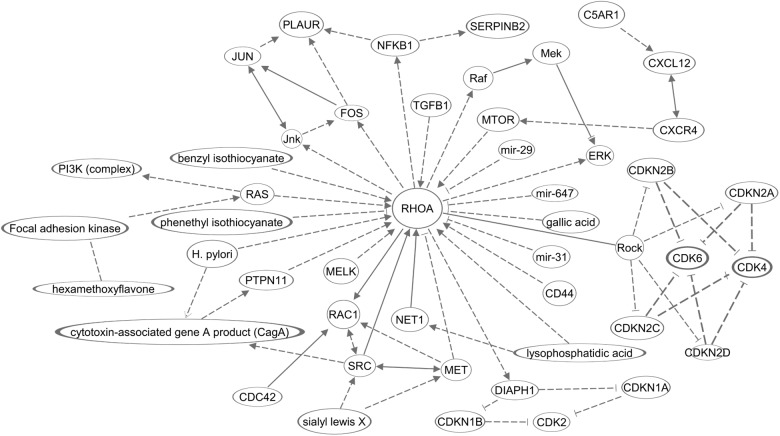
A diagram of molecular networks of RHOA in GC. In GC, regulation of RHOA described in this review was represented in the networks. Solid lines represents established regulations, and dashed lines the regulations described in this review for GC.

The molecular mechanisms in this section are summarized in Figure 2. Overall, the majority of RHOA functions, in invasion, primarily encompass cancer hallmark #2 (resistance to cell death). Other hallmarks should be investigated in future.

### Biomarker and Clinical Relevance of RHOA, and Its Molecular Regulators

*RHOA* mRNA and its protein expression have demonstrated clinical relevance, including overall survival and GC tumor stage, and in this section, we searched for RHOA clinicopathological associations out of the 63 publications in the previous section. This search revealed 10 reports (Liu et al., 2004; Pan et al., 2004; Huang et al., 2015; Chen et al., 2016; Yoon et al., 2016, 2017; Ge et al., 2017; Korourian et al., 2017a,b; Song et al., 2017) of assessment of RHOA immunohistochemical (IHC) staining and its mRNA expression (Supplementary Figure S1). In addition, manually collected studies were reviewed in this section.

For example, Huang et al. (2015) reported that the Lauren classification, diffuse subtype, significantly associated with RHOA and specific clinicopathological characteristics, while the GC Lauren classification, intestinal subtype, did not. Moreover, IHC staining of diffuse subtype GC tumors, for RHOA, associated with advanced pathological N (nodal ingress) stages and poor prognosis (i.e., disease-free and overall survival), after surgery (Huang et al., 2015; Yoon et al., 2016). In another small sample size study (Yoon et al., 2017), advanced T and TMN stages showed higher *RHOA* mRNA and its protein expression, compared to early T and TMN stages. However, these results were inconsistent with those of Korourian et al. (2017b), who reported no statistically significant difference between RHOA IHC staining and TNM stage. The inconsistency may come from clinicopathological characteristics differences (including ethnicity) and different IHC grading schemes.

Liu et al. (2004) profiled RHOA IHC staining across the sequence of GC tumor development, i.e., normal mucosa, intestinal metaplasia, dysplasia, and invasion (Liu et al., 2004). Those results showed significantly higher RHOA expression in full-fledged tumors, compared to normal mucosa, intestinal metaplasia, and dysplasia, indicating progressively increasing RHOA expression during GC development. In another study, RHOA protein expression, in a small cohort (*n* = 53), showed positive association with poor GC differentiation (Pan et al., 2004). That study also confirmed statistically significant upregulation of *RHOA* mRNA expression, in malignant GC tissues, compared to adjacent normal (Pan et al., 2004). These studies correlating poor GC differentiation status with upregulated RHOA, as determined by IHC, are consistent with two other studies (Korourian et al., 2017a,b) reporting RHOA expression to be higher in Lauren classification subtype diffuse tumors, compared to the Lauren classification intestinal subtype, with the former also positively associating with vascular invasion.

*RHOA* is also a target of miR-31, which interestingly, showed significant clinical relevance to GC (Chen et al., 2016). In that regard, RHOA IHC staining was significantly higher in tumor tissues than in adjacent normal tissues, in negative correlation with miR-31 expression (Chen et al., 2016). Another recent study (Ge et al., 2017) showed possible clinical relevance of miR-31, in that its low expression associated with advanced pathologic N stages, and positive lymphatic vessel invasion status (Ge et al., 2017). That work also indicated that such a miR-31/RHOA axis could represent a clinical marker, and possible therapeutic target, in GC.

Our recent GC study (Chang et al., 2016a) also revealed that gene expression of *RHOA* was higher in stage I GC tissue samples than in adjacent normal tissues, revealing a clinical association with early stage GC. This finding was further confirmed in an independent RHOA IHC study showing that early GC (stages T1a and T1b) tissues exhibited higher RHOA protein expression, in comparison to adjacent tissues (Song et al., 2017).

RHOA also associated with signaling by the stemness-related pathway WNT (Chang et al., 2016a), as determined by a network generation algorithm, PATHOME (Nam et al., 2014), with both pathways sharing downstream genes/proteins (i.e., “crosstalking”). It has also been shown that the Wnt pathway β-catenin degenerative complex, when inhibited by Dishevelled, allows Rho/Rac to facilitate β-catenin translocation to the nucleus (Schlessinger et al., 2009). Analogously, Wnt5a (Liu et al., 2013; Kim et al., 2017) and Wnt3a (Kim et al., 2017) were positively correlated with RHOA activation, in association with GSK3-β phosphorylation. In the brain, RHOA inhibition enhanced GSK3-induced phosphorylation and degradation of β-catenin, while also inhibiting membrane efflux by P-glycoprotein, a “stemness” phenotype (Pinzon-Daza et al., 2014). Another recent finding showed GC clinical relevance of WNT signaling (WNT5A), in terms of lymph node metastasis, Lauren classification subtype diffuse, and advanced UICC stage (Nam et al., 2017). However, clinical application of RHOA and WNT signaling (Nam et al., 2014, 2017; Chang et al., 2016a; Kim et al., 2019) yet requires functional validation, prior to assessment of therapeutic feasibility.

Currently, next-generation sequencing (NGS) data for GC patients is available in the Cancer Genome Atlas (TCGA) (Cancer Genome Atlas Research Network, 2014). We inspected *RHOA* mutations, copy number variations, and gene expression the TCGA stomach cancer dataset (258 patients) (Cancer Genome Atlas Research Network, 2014) by using cBio Portal (Gao et al., 2013). In Supplementary Figure S3, we also displayed CpG island methylator phenotype (CIMP) categories, Epstein–Barr virus (EBV) presence, microsatellite instable (MSI) status and Lauren classification along with *RHOA* genetic alterations. Somatic mutation frequency was 5.4% (14/258), and copy number alteration (CNA) frequency was 2.3% (6/258). Two out of the 14 patients having *RHOA* mutations were EBV-present. The 6 patients having CNAs were deep deletion and EBV present. Also, five out of the 6 CNA patients belonged to Lauren classification intestinal type. The TCGA created the four molecular subtypes: EBV-positive tumors, MSI tumors, genomically stable (GS) tumors, and tumors with chromosomal instability (CIN) (Cancer Genome Atlas Research Network, 2014; Zhang, 2014). According to the TCGA, genetic events of *RHOA* and Rho-family GTPase-activating proteins (GAP) were the molecular subtype GS.

## RHOA Therapeutic Implications in Gastric Cancer

### GC Therapeutics and RHOA

To date, FDA-approved drugs directly targeting RHOA remain unapproved, according to the CIViC database (Griffith et al., 2017)^1^. However, GC RHOA signaling associated with clinical efficacy of various chemotherapeutics (Kang et al., 2005; Zhou et al., 2011; Wang et al., 2015; Yoon et al., 2016; Korourian et al., 2017b) (Table 1). Roles for RHOA in chemotherapeutic efficacy were also shown in Lauren diffuse GC cells (Yoon et al., 2016; Korourian et al., 2017b). Also, in Lauren intestinal GC cell lines (AGS, SNU-638), RHOA hyperactivity reduced chemosensitivity (Kang et al., 2005; Zhou et al., 2011).

**Table 1:** RHOA involvement in actions of specific chemotherapeutics, and their descriptions.

Compounds	Description
Cisplatin (Wang et al., 2015), docetaxel (Wang et al., 2015), 5-fluorouracil (Korourian et al., 2017b), vincristine (Kang et al., 2005), taxol(Kang et al., 2005)	Non-coding RNA (miR-29, and -31) epigenetics, involved in chemotherapy efficacy, through RHOA signaling
5-fluorouracil, cisplatin (Yoon et al., 2016)	CD44(+) GC CSC cell numbers were decreased by cisplatin combined with RHOA signaling inhibitors; A cancer hallmark term, resisting cell death (cancer stem-like cells, CSCs) associated.

*Regarding RHOA, therapeutic agents, and their description are summarized in the table*.

RHOA signaling also associated with cisplatin and docetaxel therapeutic actions in GC, in addition to association with miR-29, a regulator of both catenin-δ (CTNND1) and RHOA, implicating non-coding RNA epigenetic effects of chemotherapeutic agents (Wang et al., 2015). Moreover, upregulation of miR-31 (a proposed regulator of RHOA (Mizoguchi et al., 2013), described above), enhanced chemosensitivity to 5-fluorouracil, in diffuse type GC MKN-45 cells (Korourian et al., 2017b).

Another study revealed that CD44^+^ CSCs associated with GC recurrence, following chemotherapy with 5-fluorouracil and cisplatin, in Lauren diffuse GC cells (Yoon et al., 2016). Further, combination of a potential RHOA signaling inhibitor, fasudil, with cisplatin, effectively suppressed numbers of CD44^+^ CSCs, in GC (Yoon et al., 2016). Although there is no postulated mechanism for CD44 association with RHOA activation, the Hippo-YAP signal pathway seems likely to link CD44 and RHOA, in other cell types (Zhang et al., 2014).

Regarding targeted therapies, the U.S. Food and Drug Administration (FDA) approved the HER2 antibody trastuzumab, combined with chemotherapy, for HER2^+^ GC patients (Bang et al., 2010). However, in diffuse type GC, HER2^+^ patients represent only 2–7% of the total, underscoring the crucial need for more subtype-specific therapeutics (Ushiku et al., 2016). Moreover, considering the clinical importance of RHOA in diffuse (and other) type GC (Nam et al., 2014; Chang et al., 2016a; Yoon et al., 2016; Korourian et al., 2017b; Nam et al., 2017), therapeutic development strategies remain largely undeveloped (Ushiku et al., 2016). Also recently, ramucirumab, a targeted therapy for metastatic GC, was approved by the FDA, although it is unclear whether it impacts RHOA signaling.

### Small Molecule Inhibitors for Targeting RHOA Proteins in GC

One small molecule inhibitor, Rhosin (Shang et al., 2012), inhibits RHOA signaling by directly targeting the RHOA protein, in breast and hepatocellular cancer cells (Shang et al., 2012; Lin and Zheng, 2015; Olson, 2018), as well as GC cells (Yoon et al., 2016). In our recent study, we demonstrated a hydrazide derivative, JK-122, as a new small molecule inhibitor that binds the RHOA active site, as determined using surface plasmon resonance, and was also antimitogenic toward GC cell lines (Chang et al., 2016a).

Several chemical inhibitors of RHOA signaling do not directly target the RHOA protein, but affect RHOA signaling-related proteins (Lin and Zheng, 2015; Olson, 2018). For example, CCG-1423 (Evelyn et al., 2007) is a small molecule that suppresses RHOA signaling (Evelyn et al., 2007) by binding to the phosphatase and actin regulator RPEL, to inhibit RHOA/MKL/SRF signaling (Hayashi et al., 2014). While unsuitable for translational development, these compounds provide proof-of-concept for experimental investigation of RHOA (and its other family members’) signal inhibition (Olson, 2018), as the identification of therapeutic small molecule inhibitors, that selectively bind RHOA, remain urgently needed.

## Conclusion

Here, we reviewed literature of how RHOA’s roles, in gastric cancer (GC), associate with the second-generation cancer hallmark term, “activation of invasion and metastasis” (Kim et al., 2008; Chen et al., 2012; Du et al., 2014). Two other cancer hallmarks (“resistance to cell death” and “sustainment of proliferative signaling”) also were linked to studies of GC. Cancer stem-like cells, well known inhabitants of the tumor microenvironment, have been also implicated in GC (Yoon et al., 2016). Thus, molecular and biochemical studies of RHOA, as related to other cancer hallmarks, need to be performed in the future.

RHOA expression in GC, as reviewed in the literature, shows clinical association with Lauren diffuse subtype GC, and may have potential prognostic value (Nam et al., 2014, 2017; Chang et al., 2016a; Yoon et al., 2016; Korourian et al., 2017b). However, in terms of GC therapeutic options, small chemical inhibitors that directly bind RHOA, have yet to translated to GC patients. However, our recent study (Chang et al., 2016a) showed that RHOA activity could be regulated by specific small chemicals that bind the protein, indicating RHOA to be a druggable target in GC. For facilitating therapeutic discovery in GC, GC *in vitro* models should be continuously developed (Chang et al., 2016b). In summary, although existing evidence demonstrates the feasibility of employment of RHOA as both a biomarker candidate and druggable target, further investigation of its application to GC (and other cancer) therapy, is urgently needed.

## Author Contributions

SN supervised the study and drafted the manuscript. All authors procured and reviewed publications suitable for this review article, and read and approved the manuscript.

## Conflict of Interest Statement

The authors declare that the research was conducted in the absence of any commercial or financial relationships that could be construed as a potential conflict of interest.

## Footnotes

1 civicdb.org
