# Supplementary material for: RHOA in Gastric Cancer: Functional Roles and Therapeutic Potential
Source: Front Genet. 2019 May 15;10:438. doi: 10.3389/fgene.2019.00438 (PMC6529512; doi:10.3389/fgene.2019.00438)
Supplement: Supplementary file 1 [file Data_Sheet_1.PDF]

RHOA in gastric cancer: functional roles and therapeutic potential

Supplementary Table 1.
Supplementary Figures 1 through 3.

Supplementary Table S1. The GC cell line characterization. Patient information, ethnicity, histology, Lauren classification, culture site, therapy record, *RHOA* mutation status, and RHOA immunohistochemistry in xenograft were summarized (Modified from the Table S1 of Chang et al (Chang et al., 2016)).

GC cells	Patient (age range)	Ethinicity	Prior therapy	Lauren classificatioin	Site	Histology	Cancer cell line culture site	*RHOA* mutation[+](Barretina et al., 2012)	RHOA IHC in xenograft [¶](Chang et al., 2016)
NCI-N87	NA	American	Cytotoxic therapy	ND	NA	Well differentiated adenocarcinoma	Primary tumor/liver metastatsis		Low
SNU-668	> 60	Korean	None	ND	NA	Signet-ring cell carcinoma	Ascites		-
NUGC-4	NA	Japanese	NA	ND	NA	Signet-ring cell carcinoma	NA		-
NCC-19	> 50	Korean	None	Intestinal	Body	Moderately differentiated tubular adenocarcinoma	Primary tumor		Medium
MKN-45	> 60	Japanese	NA	Diffuse	NA	Poorly differentiated adenocarcinoma	Liver metastasis		Low
NUGC-3	NA	Japanese	NA	ND	NA	Poorly differentiated adenocarcinoma	NA		-
SNU-1967	> 40	Korean	None	Diffuse	High body	Poorly differentiated adenocarcinoma	Ascites		Low
MKN-28	> 70	Japanese	NA	Intestinal	NA	Moderately differentiated tubular adenocarcinoma	Metastatic foci to lymph nodes		-
SNU-638	> 40	Korean	None	ND	NA	Poorly differentiated adenocarcinoma	Ascites		-
MKN-1	> 70	Japanese	NA	Diffuse	NA	Adenosquamous carcinoma	Primary tumor		-
SNU-601	> 30	Korean	FAM, 5-FU+cisplatin	ND	NA	Signet-ring cell carcinoma	Ascites		High
SNU-5	> 30	Korean	FAM	ND	NA	Poorly differentiated tubular adenocarcinoma	Ascites		-
SNU-16	> 30	Korean	None	ND	NA	Poorly differentiated adenocarcinoma	Ascites	ENST00000418115.1 // p.R5W // p.F39L	-
NCC-59	> 60	Korean	None	Intestinal	Antrum	Moderately differentiated tubular adenocarcinoma	Ascites		-
SNU-484	> 50	Korean	None	ND	NA	Poorly differentiated adenocarcinoma	Primary tumor		High
SNU-719	> 50	Korean	None	ND	NA	Moderately differentiated adenocarcinoma	Primary tumor	ENST00000418115.1 // p.A61V	-
IM95M	> 60	Japanese	NA	ND	NA	Moderately differentiated adenocarcinoma of stomach	NA	ENST00000418115.1// p.P111S	-
SNU-216	> 40	Korean	None	ND	NA	Moderately differentiated adenocarcinoma	Lymph node metastasis		-
NCC-20	> 50	Korean	None	ND	Antrum	NA	Ascites		-
NCC-	> 40	Korean	None	Diffuse	Antr	Signet-ring cell	Primary		-

24					um	carcinoma	tumor		
AGS	> 50	Caucasian	NA	ND	NA	Moderately-poorly differentiated adenocarcinoma	Primary tumor	ENST00000422781.1 // p.E64del	-
MKN-74	> 30	Japanese	NA	Intestinal	NA	Moderately differentiated tubular adenocarcinoma	Liver metastasis		-
SNU-1	> 40	Korean	None	ND	NA	Poorly differentiated tubular adenocarcinoma	Primary tumor		-
SNU-620	> 50	Korean	None	ND	NA	Poorly differentiated adenocarcinoma	Ascites		-
SNU-1750	> 60	Korean	None	Diffuse	Antrum	Poorly differentiated tubular adenocarcinoma	Primary tumor		-

NA=Not Available; ND=Not Determined

[+]Mutation of RHOA was obtained from Broad Institute Cancer Cell Line Encyclopedia (CCLE) (Barretina et al., 2012).

[¶]IHC score: 2.5~3.0 as high; 2.0~2.5 as medium; less than 2.0 as low (Chang et al., 2016)

Supplementary Figure S1. Publication selection procedure for RHOA in GC. The bases of sections 2.1 and 2.2 were selected by following the procedure.

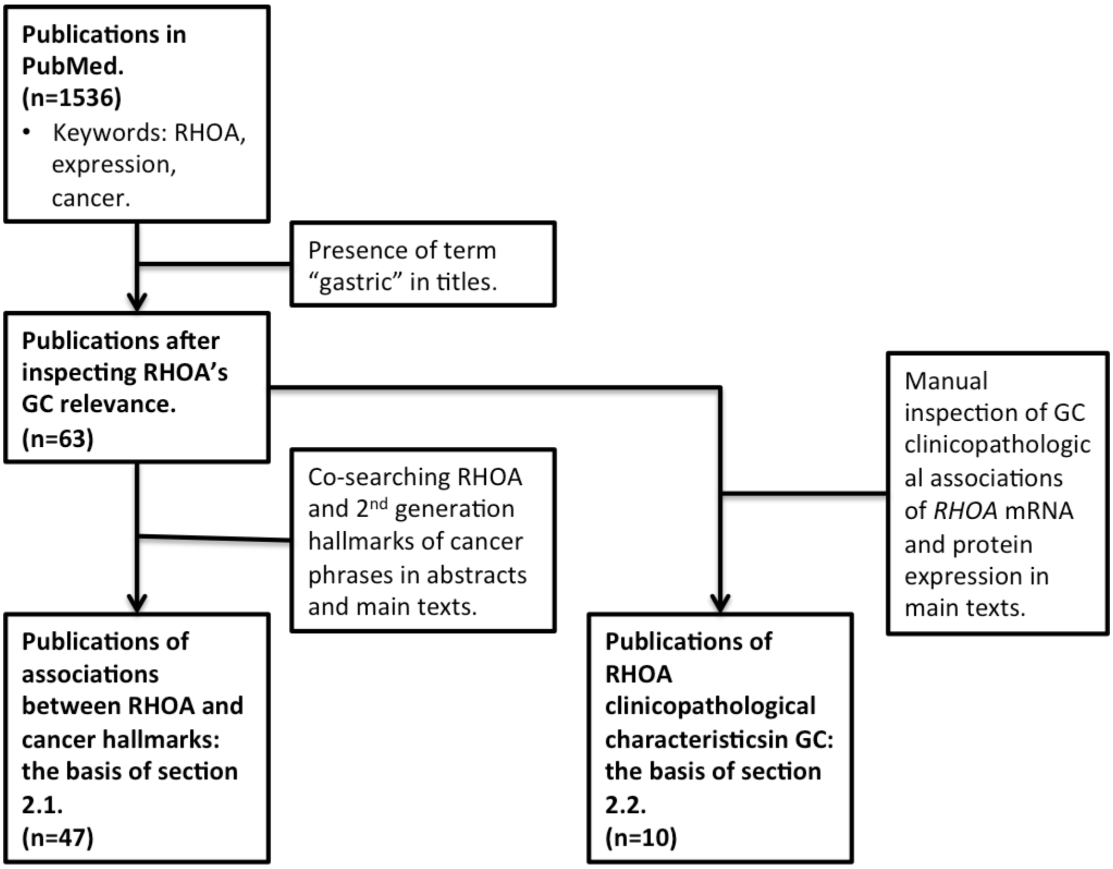

Supplementary Figure S2. RHOA-related gastric cancer (GC) article assignment to specific cancer hallmarks. Functional assignment of the RHOA-related GC articles, to second-generation cancer hallmarks (Hanahan and Weinberg, 2011), and the numerals in parentheses indicate the number of publications. (N/A: no publications assigned in the hallmark terms)

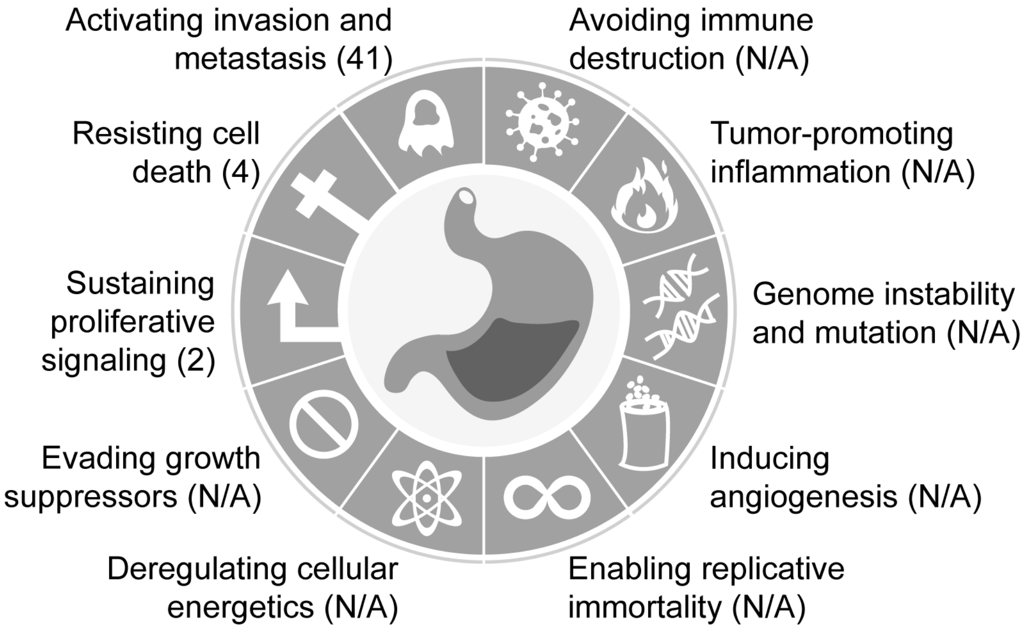

Supplementary Figure S3. Genetic alterations of RHOA of 258 GC patients in TCGA.

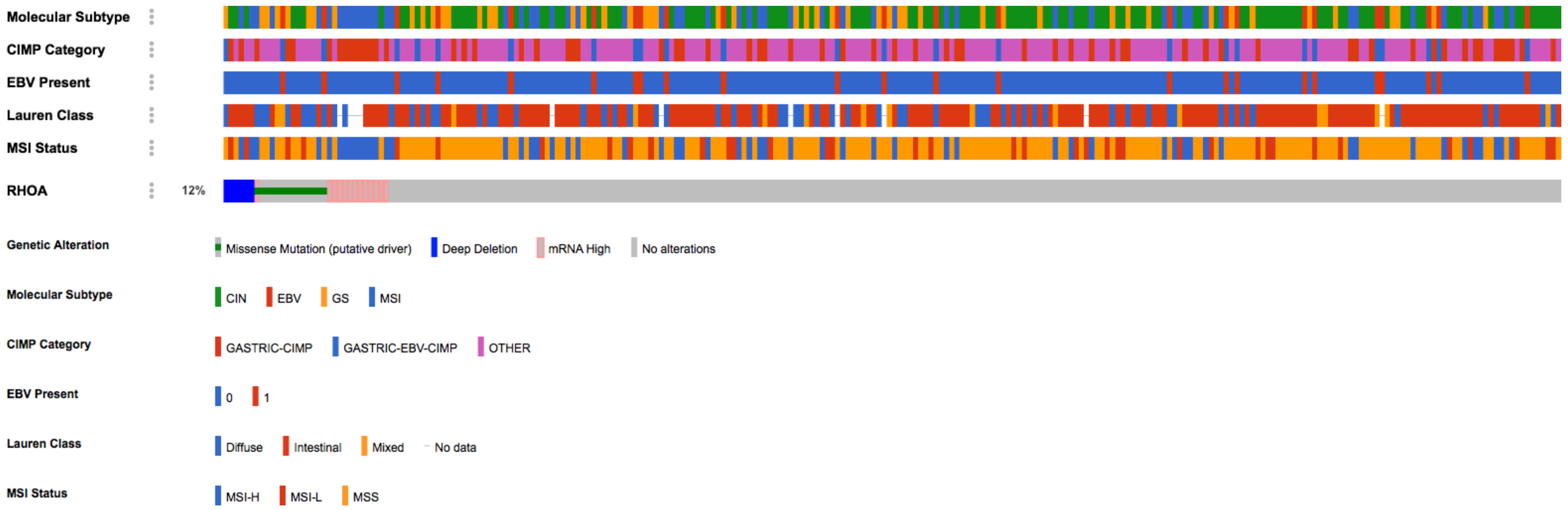

References

Barretina, J., Caponigro, G., Stransky, N., Venkatesan, K., Margolin, A.A., Kim, S., et al. (2012). The Cancer Cell Line Encyclopedia enables predictive modelling of anticancer drug sensitivity. *Nature* 483(7391), 603-607. doi: 10.1038/nature11003.

Chang, H.R., Park, H.S., Ahn, Y.Z., Nam, S., Jung, H.R., Park, S., et al. (2016). Improving gastric cancer preclinical studies using diverse in vitro and in vivo model systems. *BMC Cancer* 16, 200. doi: 10.1186/s12885-016-2232-2.

Hanahan, D., and Weinberg, R.A. (2011). Hallmarks of cancer: the next generation. *Cell* 144(5), 646-674. doi: 10.1016/j.cell.2011.02.013.
